# Inherited Bleeding Disorders in Iraq and Consanguineous Marriage

**Published:** 2018-10-01

**Authors:** Nidal Karim Al-Rahal

**Affiliations:** The National Center of Hematology, Al-Mustansiriyah University, Baghdad, Iraq

**Keywords:** Consanguinity, Inherited bleeding disorders, Von Willebrand disease

## Abstract

**Background: **Consanguineous marriage is defined as inbreeding between second cousins or closer. In such families there will be a potential increase in the autosomal recessive traits with its lethal effect, with an increased risk of morbidity and mortality in the new generation. Inherited bleeding disorders (InBDs) are rare complicated diseases, difficult and expensive to treat, the defect usually due to quantitative or qualitative deficiency of clotting factors, platelets or fibrinolysis. This study attempts to assess the diversity, the frequency and the clinical features of inherited bleeding disorders (InBDs) in central part of Iraq and to determine the state of consanguineous marriage.

**Materials and Methods: **This is a prospective cross-sectional study conducted in the National Center of Hematology NCH, Baghdad, Iraq between June2014 and June 2017. In total, 256 pediatrics and adult patients were included. Full bleeding history, family history, drug history and consanguineous marriage were recorded and followed by medical examination. First-line laboratory tests were performed and then were followed by further tests included mixing study, lupus anticoagulant testing, clotting factor activity assay, von Willebrand Antigen (VW: Ag), Ristocetin co factor vWF: RiCoF activity and platelet function test.

**Results** **:** The range of age was from 1 month to 57 years, with mean age 8.424±8.623 years and median age of 6.5years. The male to female ratio was 1.1:1. The most common age group was in the range of 1-10 years (46.45%). Family history was positive in 55.07% of patients (P >0.05). The consanguinity was found in 76.95% of the families studied (P <0.0001). The most prevalent InBD was von Willebrand disease (42.98%) with majority type 3VWD (86.4%). The second most prevalent was thrombasthenia (36.71%) and the majority had Glanzmann’s thrombosthenia (86.2%). Rare bleeding disorders (RBDs) were observed in 6.25% of patients and the most common factor deficiency was FVII.

**Conclusion: **Consanguinity is high in patients with inherited bleeding disorders in Iraq, leading to emergence of life-threatening autosomal recessive inherited diseases. Genetic counselling is recommended besides education and awareness to minimize such rare illnesses in the community.

## Introduction

 Consanguineous marriage is defined as inbreeding between second cosines or closer^1^. All over the world 20% of human prefer consanguineous marriage and 8.5% of children have relative parents ^2^. In such families, there will be increase in the autosomal recessive traits with an increased risk of morbidity and mortality in the new generation, which is a social and medical problem^3^^,^^4^. Inherited bleeding disorders (InBDs) manifest itself as lifelong bleeding diseases. In severe cases, they are typically diagnosed in early childhood. They present with easy bruising with or without trauma; the defect is usually due to quantitative or qualitative deficiency of clotting factors, platelets or fibrinolysis^5^. Peyvandi F et al. ^6^ from Italy concluded that the prevalence of RBDs ranges from approximately 1 in 2 million to 1 in 500 000, being higher in countries where consanguineous marriages are diffused. Consanguinity is high in communities in the Middle East, it was about 26% in 1988^7^. According to Pakistan Demographic and Health Survey (DHS), two thirds of marriages in Pakistan were consanguineous^8^.The most common 3 inherited bleeding disorders are von Willebrand disease with hemophilia A and hemophilia B, accounting for 95-97% of InBDs; while rare bleeding disorders (RBDs) account for 5%. Rare bleeding disorders include fibrinogenemia, deficiency of actor (F) II. V. VII. XI, XIII, combined FV and VIII and vitamin K-dependent coagulation factors (VKDC). Like Iran, there is a significant association between FXIII Val34Leu polymorphism and unexplained recurrent pregnancy loss^10^. Rare diseases of platelet function defects (PFD) include a large group of illnesses with high prevalence in Middle East, India and developing countries^11^. It is well accepted by many authors that VWD is the most prevalent InBD in patient population with a prevalence of 1-2% in adults ^12^ and children^13^.

Despite the impact of InBDs, the available data are scanty in Iraq. Therefore, this study was conducted with the aim of studying the magnitude, diversity, and the clinical features of different inherited bleeding disorders, primarily in central Iraq and determining the state of consanguineous marriage.

## MATERIALS AND METHODS

 This is a prospective cross-sectional study conducted in the National Center of Hematology NCH, Baghdad, Iraq between June 2014 and June 2017. All pediatrics and adult patients suspected to have inherited bleeding disorders were entered into the trial. The current study was approved by the Ethics Committee of the institution and was conducted in accordance with Helsinki’s declaration^14^. Informed consent was obtained from all patients, the parents or caregivers. Inclusion criteria: all patients: neonates, children and adults with bleeding tendency suspected to have InBD, some patients with positive family history of a similar. Patients with acquired hematological illness leading to bleeding tendency such as immune thrombocytopenia (ITP), aplastic anemia, malignancies, disseminated intravascular coagulation (DIC) and patients on anticoagulant drugs were excluded. 

Full bleeding history, age of onset of bleeding, site , extent, duration, number, its relation to trauma or after interventions, admission to hospital, history of blood transfusion or its products, family history , drug history and consanguineous marriage were recorded and followed by medical examination. Grading of the patients regarding bleeding severity was assessed according to the European Network of Rare Bleeding Disorders (EN-RBD) 2012^15^. Patients were then divided into 3 categories according to their bleeding episodes, the location of the bleeding and its clinical impact and whether it was spontaneous or post-traumatic. Grade I: post-traumatic bleeding or after medication (anti-platelet) injection, Grade II: spontaneous minor bleeding including bruises, ecchymosis, minor wounds, epistaxis, oral cavity bleeding and menorrhagia and Grade III: hemarthrosis, hematoma, central nervous system (CNS) bleeding, gastrointestinal (GI) bleeding and umbilical bleeding. 

**Figure 1:**
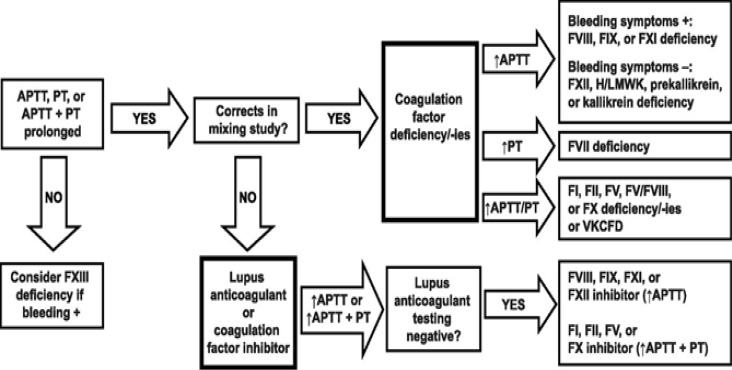
Diagnostic flow chart of inherited bleeding disorders (Suchitra S. Acharya) ^16^

First-line laboratory tests included complete blood count with blood film Hemolyzer 5 (Analyticon), prothrombin time (PT) normal value 13-16 sec. (AST, Stago, Asniẻres-sur-Seine, France), activated partial prothrombin time (APPT) normal value 30-40 sec. (AST, Stago, Asniẻres-sur-Seine, France) by using semi–automated, bench-top Hemostasis coagulation analyzer (Diagnostica Stago, model: ST ART, France), blood group. Bleeding time (BT) was determined by Ivy’s method. Further investigations were performed as per the requirements. The diagnostic flow chart of IBDs which was followed and regarded as a study design for the diagnosis of patients with IBD is shown in Figure 1^16^.

The investigations included mixing study, lupus anticoagulant testing and clotting factor (F) activity assay by one-stage coagulometric method normal value (50-150%) (AST, Stago, Asniẻres-sur-Seine, France), except for F XIII, the diagnostic test was solubility test. Von Willebrand Antigen (VW: Ag) by Eliza technique (normal value 50-150%) (AST, Stago, Asniẻres-sur-Seine, France), Ristocetin co factor (VW: RCoF) activity (normal value ≥45%), and platelet function test by light transmission aggregometry LTA {Platelet Aggregation Profile (PAP-E8), Bio/DATA corporation, USA Data}. 

As illustrated in the diagnostic flow chart, If APTT, PT or APTT+PT were prolonged, mixing study was performed and if it was correctable, coagulation factor assays were performed accordingly. In cases with prolongation of APTT, FVIII, FIX and FXI assays were conducted. But, if there was only prolongation of the PT, FVII assay was performed. In cases which both APTT and PT were prolonged, we assessed the following rare factors: FI, FII, FV, FX, combined FV/FVIII or vitamin K deficient factors: X, IX, VII, and II. In patients with APTT, PT or APTT+PT were prolonged and mixing study was not correctable, thus lupus anticoagulant was performed; if it was negative, coagulation factor, inhibitors were suspected. In cases with mixing study that was not correctable with prolonged APTT, FVIII, FIX, FXI or FXII and inhibitors were assessed. VWD was classified to type 1, 2 or 3 according to the International Society of Thrombosis and Haemostasis (ISTH) classification (revised in2006)^17^. Since VWF is an acute phase reactant, and it is affected by stress, exercise, pregnancy, contraceptive pills and corticosteroid therapy, so evaluation of any patient under strong suspicion was repeated up to 3 times, taking into consideration the blood group O patient have a lower normal values of VW: Ag and VWF: RCo activity^17^.

The collected data were analyzed using SPSS version 18.0. The mean of the variables was determined while comparison of the variables was estimated applying Chi-square. A P-value ≤0.05 was considered statistically significant. 

## Results

 Two hundred fifty-six patients enrolled in this study suspected to have InBDs. The range of age was from 1 month to 57 years, with mean age of 8.424±8.623 years. The median age was 6.5 years. The study consisted of 136(53.12%) male patients and the male to female ratio was 1.1:1. The most common age group was in the range of 1-10 years (64.45%), followed by age group 11-20 years (17.18%). Family history of a similar bleeding disease was found in 55.07% of the patients; (P-value >0.05).The parents of 197(76.95%) patients had consanguineous marriage ;( P- Value <0.0001). First cousin marriage was the commonest185 (72.4%). The majority of our patients were from Baghdad (n=110, 42.96%), followed by provinces south of Baghdad (n=98, 38.28%), including Karbala, Najaf, Babylon, Dewania, Muthana and Basra (Table 1).

**Table 1:** The demographic characteristics of patients with IBD

Parameter (No.= 256)		No. (%)
Gender	Male	136(53.12%)
	Females	120(46.88%)
Age group: years	Birth-1	22(8.59%)
	1-10	165(64.45%)
	11-20	44(17.18%)
	≥20	25(9.76%)
Family history	Positive	141(55.07%)
	Negative	115(44.93%)
Consanguinity	Positive	197(76.95%)
	Negative	59(23.05%)
Residency	Baghdad	110(42.96%)
	South	98 (38.28%)
	East	21(8.20%)
	North	17 (6.64%)
	West	10 (3.90%)

Analyzing the current data of patients (n=256) showed that von Willebrand disease (n=110, 42.98%) type 3(n=95, 86.4%) was the most common InBD. There were only 3 patients with type1 and 8 patients with type 2 VWD (of whom 6 were diagnosed with type 2 A or 2M). The author was unable to differentiate between type 2 A and M VWD since the chromatography test only detected high molecular weight multimers (HMWM). The remaining 4 patients were diagnosed with pseudo- VWD (Table 2).

**Table 2:** Distribution of types of deficiencies in patients with inherited bleeding disorders

** Type of deficiency No.= 256**		**No.**
VWD 110(42.98%)	Type 3 VWD	95
	Type 2 A or M VWD	6
	Type 2B VWD	2
	Type 1 VWD	3
	Pseudo VWD	4
Thrombosthenia(PFD) 94(36.71%)	GT	81
	BSS	5
	Other PFD	8
HA 33(12.89%)	Severe	21
	Moderate	4
	Mild	8
HB 3(1.17 %)	Severe	1
	Mild	2
RBD 16(6.25%)	F VII	7
	F XI	2
	A fibrinogenemia	1
	F V	1
	F X	1
	F XIII	3
	Combined V&VIII	1

The second most common type of InBDs was thrombasthenia 94(36.71%). Of whom, 81(86.2%) patients had Glanzmann’s thrombasthenia (GT), and Bernard Solier syndrome (BSS) was found in 5 patients. In addition, 8 patients with other rare types of thrombasthenia were observed, but proper diagnosis was not feasible due to lack of sophisticated investigations.

Hemophilia A (HA) was found in only 33 (12.89%) patients; the majority of whom (n=21) had severe type with FVIII activity level <1%, 4 had moderate with FVIII activity level (1-5%), and 8 patients had mild HA with FVIII activity level of (6-40%). 

FIX deficiency was found in 3(1.17%) patients, of whom 1 had severe factor level activity and 2 had mild HB. Among 16 (6.25%) patients with rare bleeding disorders (RBDs) , 7, 3 and 2 patients had F VII ,F XIII, F XI deficiency, respectively. The remaining 4 patients had other RBDs such as FI, FV, F X and Combined V&VIII, (Table 2). 

**Figure 2:**
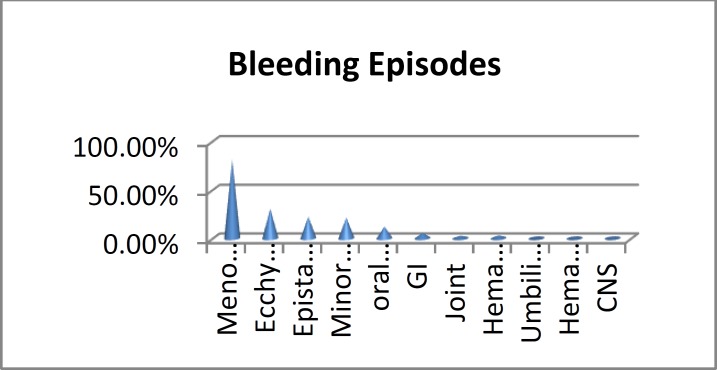
Bleeding episodes in patients IBD

With respect to different types of bleeding episodes in our patients, the most common type of bleeding was ecchymosis(30.6%), followed by epistaxis (22.3%), minor wound (21.5%) and oral bleeding (12.1%) (Table 3) (Figure 2). 

**Table 3:** Spectrum of bleeding episodes in patients with IBD

**Type of Bleeding**	**No. Total 480 (%)**
Ecchymosis	147 (30.6%)
Epistaxis	107 (22.3%)
Minor wound	103 (21.5%)
Oral bleeding	58 (12.1%)
Menorrhagia	30/37 (81.1%)
GI	27 (5.6%)
Joint	13 (2.7%)
Hematoma	15 (3.1%)
Umbilical	3 (0.6%)
Hematuria	5 (1%)
CNS	2 (0.4%)

Menorrhagia, the most common presentation in females, was observed in 30 girls and women. Out of 37 (81.1%) patients, 17 were diagnosed with type3 VWD, 8 with GT, and 2 with F VII deficiency. The 3 remaining female patients were diagnosed with BSS, type 2B VWD and type (2 A or M) VW D, respectively. Furthermore, all patients affected by menorrhagia had iron deficiency anemia, of whom half had a history of blood transfusion. Figure 2 visualized the spectrum of bleeding in patients with InBDs. Hemarthrosis was observed in 13 patients (2.7%), of whom 8 had severe HA, 1 had severe H B and 4 had type 3 vWD, respectively. Patients with type 3vWD were labeled incorrectly as having hemophilia. 

Regarding bleeding severity, 58(61%) patients with type3 vWD grade II experienced spontaneous mild bleeding (ecchymosis, minor wounds, bleeding of the oral cavity, epistaxis and menorrhagia), whereas 37(39%) of cases of type 3 vWD showed severe manifestations of grade III (spontaneous major bleeding). 

 Similarly, the majority of patients with GT (n=65, 80.2%) had grade II and 16(19.8%) had grade III bleeding severity. On the contrary, the majority of patients with severe Hemophilia (n=16, 76.2%) experienced severe bleeding (grade III) with hemarthrosis. While most of the cases of FVII deficiency (4 out of 7 cases) had grade II bleeding severity, 2 cases had grade III bleeding severity with hemarthrosis, hematuria and CNS bleeding. Similar to the patient with fibrinogenemia, 2 out of 3 patients with factor FXIII deficiency had grade III bleeding severity (umbilical bleeding). Other patients with RBDs including FV, FX, FXI and combined FV and VIII had grade II bleeding severity (Table 4).

**Table 4:** Grading of bleeding severity in patients of IBD

**Type of deficiency No.(256)**		**No.**	**Grade I**	**Grade II**	**Grade III**
VWD 110(42.96%)	Type 3 VWD	95		58(61%)	37(39%)
	Type 2 A or M VWD	6		5	1
	Type 2B VWD	2		2	
	Type 1 VWD	3		3	
	Pseudo VWD	4		4	
Thrombosthenia 94(36.71%)	GT	81		65(80.2%)	16(19.8%)
	BSS	5		5	
	Other PFD	8	3	3	2
HA 33(12.89%)	Severe	21		5(23.8%)	16(76.2%)
	Moderate	4		2	2
	Mild	8	4		
HB 3(1.17%)	Severe	1			1
	Mild	2	2		
RBD 16(6.25%)	F VII	7	1	4	2
	F XI	2		2	
	F V	1		1	
	F X	1		1	
	A fibrinogenemia	1			1
	F XIII	3		1	2
	Combined V&VIII	1		1	

## Discussion

 Consanguinity is the union between couples with at least one common ancestor^18^, while in genetics it is defined as the union of two individuals who are second cousin or closer^19^. Matting between close biological kin is discouraged in western countries, but in North Africa, large part of Asia and Middle East still remain preferable. However, information regarding the prevalence of different InBDs and consanguinity is lacking in Iraq. Accordingly, this study establishes the prevalence of different types of InBDs and consanguinity in Iraq. There is a positive association between consanguineous marriages (CM) and pediatric morbidity by expression of detrimental recessive genes such as retinal dystrophies, deafness, mental and developmental disabilities ^20^^,^^21^, complex congenital heart lesions, besides thalassemia and other hematological disorders^22^^,^^23^^,^^24^. As a result of CM, the rare autosomal recessive diseases run more commonly in close families such as type 3 VWD and thrombasthenia. Both have recessive inheritance ^25^. This concept was proved in the current study as the most prevalent InBD in our patients was VWD (n=110, 42.98%) and the majority of cases were type 3VWD (n=95, 86.4%). The second most common disease was thrombasthenia (n=94, 36.71%) mainly GT (n=81, 86.2%). The male to female ratio (1.1:1) in patients (n=256) enrolled in the study was nearly equal. The vast majority of patients with autosomal recessive inheritance pattern as type 3 VWD, GT and other rare bleeding disorders (RBD) (n=16, 6.25%) showed that 85.94% of our patients had autosomal recessive InBDs. It means that consanguineous marriage is still high (n=197, 76.95%), especially among first cousins (72.4%). The most common age group consisted of 165 (46.45%) patients in the age range of 1-10 years, which means that the parents or the care givers seek medical advice early because the majority of our patients had Grade II bleeding severity. In nearby countries like Saudi Arabia, a high prevalence of CM (52.0%) was seen in Dammam province ^26^ where the highest *frequency* of first *cousin marriages* occurred (39.3%). Also, in Saudi Arabia, in Riyadh province, the prevalence of CM was 51.3 %^27^. The frequency of CM was estimated as 35.5% in Lebanon where the highest prevalence of first -cousin marriage (31.6%)^7^ was seen. Meanwhile, it was 28.4% among the Shiites in Lebanon^19^. In Syria, the total rate of CM was 35.4%^18^, but a study from Oman showed higher prevalence of CM (52%) and stated that first-cousin marriage constituted 39% of the marriages ^28^. Also, a report from Sana´a Yemen showed a high prevalence of CM 44.7%^29^. In Pakistan, several studies confirmed the high prevalence of CM, accounting for 38 to 59 % of marriages ^30^^,^^31^, while, in Kashmir, it was 62 % ^32^. The overall rate of CM in Iran was 38.65 and first-cousin union was the most common, accounting for 27.9% of marriages^33^. The prevalence of CM in Arab communities and other Muslim countries like Pakistan and India is higher than in European, South and North American, Eastern Asia, Oceanic and South African countries^34^. Analysis of data in the current study showed strong association between autosomal inherited bleeding disorders and consanguinity. In this study, 85.94% of our patients had autosomal recessive IBDs and the prevalence of consanguineous marriages (CM) was found 76.9% (n=197). Moreover, the majority of the marriages were between first cousins (72.4%). Ahmed et al. ^35^ from India studied 1576 cases with IBDs and found that RBDs such as thrombasthenia had higher frequency (27.77%) in comparison to FIX. Similarly, in the current study, thrombasthenia and FIX deficiency were found in 36.71% and 1.17% of patients, respectively. A study conducted on IBDs in Chandio tribe in Pakistan revealed a high prevalence of autosomal InBDs type3 VWD (51.02%) and thrombasthenia (48.98%) with no case of hemophilia A or B^25^. Peyvandi F et al. ^6^ from Italy concluded that the prevalence of RBDs ranging from approximately 1 in 2 million to 1 in 500 000, being higher in countries where consanguineous marriages are diffused. Recessively transmitted InBDs are so common in Muslim countries and south India where CM is common, so that their prevalence can surpass the prevalence of hemophilia B, which is indeed an impotent clinical and social problem^4^. Mannucci PM et al.^9^ agreed that there was an increased prevalence of RBDs in communities with high consanguinity union. Von Willebrand disease has a prevalence of 1% with predominance of type 1 VWD^17^, but many researches from the East demonstrated higher frequency of type 2 and type 3 VWD^35^^,^^3^. Similarly, in the current study, type 3 VWD was the most common type of VWD (86.4%) among all cases of VWD. One of the explanations for this high figure of type 3VWD is that those patients are severe bleeders, so they seek medical advice earlier than type 1VWD; moreover, cases of type1 VWD are milder bleeders and might be miss diagnosed or were not referred for proper diagnosis. Cases of VWD are classified according to the criteria recommended by the VWD Subcommittee of ISTH (International Society of Thrombosis and Hemostasis) first published in 1994 and then revised in 2006 ^17^. Type 3 VWD with complete deficiency of VWF is rare (1:250,000 to 1:1,000,000), inherited as autosomal recessive. Type 3VWD is diagnosed by having undetectable level of VWF activity, usually prolonged bleeding time and very low level of FVIII (1-5) IU ^37^. 

Regarding severity of bleeding in patients with type 3VWD, 61% had grade II (spontaneous minor bleeding) bruising, ecchymosis, minor wounds, bleeding of the oral cavity, epistaxis, and menorrhagia. Similar results have been reported by many authors^38^^,^^39^. Menorrhagia was the most common manifestation in 17 (89.5%) adolescent and adult females with type 3 VWD. Many literatures estimated that the frequency of VWD in females with menorrhagia can reach up to 5-20%^36^^,^^40^. While grade III bleeding severity (spontaneous major bleeding) was observed in 39% of patients with type 3VWD such as GI bleeding ^5^^, ^^24^.

The second most common InBDs found in the current study were thrombasthenia and GT in 94(36.71%) and 81 patients, respectively. The majority of GT casas (80.2%) had grade II bleeding severity and 19.8% experienced severe bleeding manifestations of rade III. Thrombasthenia or platelet function defect (PFD) disorders are a group of rare autosomal inherited disease most prevalent in communities with high consanguinity in Middle East, developing countries and India^5^^,^^9^^,^^11^. In GT, there is a quantitative and /or qualitative abnormality of platelet fibrinogen receptor, glycoprotein IIb/IIIa which is mandatory for platelet aggregation^41^. Awidi AS et al.^42^ from Jordon also found GT as the second most common IBD. A study conducted by Eman A. et al. ^43^ in Egypt and Saudi Arabia showed GT as the third most common IBD. Several studies in India, similar to ours, demonstrated high prevalence of thrombosthenia due to same causal factor of high prevalence of consanguinity^35^^,^^36^^,^^44^. In a study conducted by Munira B et al.^25^ in a tribe in Pakistan, due to the strong consanguinity, only two inherited bleeding disorders including VWD (51.02%) and thrombasthenia (48.98%) were found. Of whom, 10% with platelet function defect had GT and no case of hemophilia was reported. Additionally, type 3 VWD (13.27%) was more common than type 1 and 2 VWD (11.22%). On the contrary, as demonstrated by the World Federation of Hemophilia(WFH) global report of 2012^45^, in developed countries such as Canada where the rate of CM is low, the most common InBD is type1 VWD (n=4669, 46.53%), followed by hemophilia (n=4023,40.09%). Moreover, Thrombasthenia with all RBDs (n=1341) accounts for only 13.36% of patients. Hayward CPM et al.^11^ concluded that thrombasthenia, particularly GT, has increased incidence in regions with high consanguinity. 

Rare bleeding disorders (RBDs) were observed in 16(6.25%) participants. Like the results found in Jordan^42^ and Iran^46^, the most common factor deficiency was FVII in patients (n=7). Similar to the study conducted by Peyvandi et al.^47^, most of the cases of FVII deficiency (4 out of 7 cases) had grade II bleeding severity; 2 cases had grade III bleeding severity and only 1 case had CNS bleeding. Similar to the patient with fibrinogenemia, 2 out of 3 patients with factor FXIII deficiency had grade III bleeding severity (umbilical bleeding).

Other patients with RBDs including FV, FX, FXI and combined FV and VIII had grade II bleeding severity. Rodeghiero F ^48^ stated that the most severe bleeding is found in fibrinogenemia, FX and FXIII deficiencies. Peyvandi F^47^ concluded that umbilical cord bleeding typically seen in FXIII and fibrinogenemia is often found in other rare factor deficiencies: FII, FV and FX. Similarly, in this study, it was seen in 2 out of 3 patients with FXIII deficiency and one patient with fibrinogenemia. Several approaches recommended by World Health Organization (WHO) to minimize the deleterious effects of consanguinity on families and their children are: identifying families at risk of having genetic illnesses, providing genetic counselling, detecting carriers at risk, education, awareness and giving advice to reduce intermarriages between carriers at risk if testing for carriers is not available. 

## CONCLUSION

 Consanguinity is high in patients with inherited bleeding disorders in Iraq, leading to emergence of life-threatening autosomal recessive inherited diseases. Genetic counselling is recommended besides education and awareness to mitigate and minimize such rare illnesses.
